# E-Scooter‑, E-Bike- und Fahrradverletzungen im gleichen Zeitraum – eine prospektive Vergleichsstudie eines Level-1-Traumazentrums

**DOI:** 10.1007/s00113-021-01136-x

**Published:** 2022-01-14

**Authors:** Heinz-Lothar Meyer, Max Daniel Kauther, Christina Polan, Benedikt Abel, Carsten Vogel, Bastian Mester, Manuel Burggraf, Marcel Dudda

**Affiliations:** 1 Klinik für Unfall‑, Hand- und Wiederherstellungschirurgie, Universitätsklinikum Essen, Hufelandstraße 55, 45147 Essen, Deutschland; 2 Klinik für Unfallchirurgie und Orthopädie, Kinderorthopädie, Agaplesion Diakonieklinikum Rotenburg, Rotenburg, Deutschland

**Keywords:** Alternatives Beförderungsmittel, Pedelec, Elektrofahrzeug, Kopfverletzungen, Helm, Alternative means of transport, Pedelec, Electric vehicle, Head injuries, Helmet

## Abstract

**Hintergrund:**

Die tatsächliche Anzahl verunglückter E‑Scooter-Fahrer in Deutschland scheint deutlich höher zu sein, als es die aktuellen Zahlen des Statistischen Bundesamtes vermuten lassen. Diese epidemiologische Studie untersucht E‑Scooter-Verletzungen und vergleicht diese mit E‑Bike- und Fahrradverletzungen.

**Fragestellung:**

Zur Schaffung einer vergleichbaren Datenlage über die Gefahren von E‑Scootern, E‑Bikes und Fahrrädern wurden die typischen Verletzungsmuster analysiert und daraus Präventionsmöglichkeiten abgeleitet.

**Material und Methode:**

Es wurden alle Patienten, die sich nach Unfällen mit Beteiligung von E‑Scootern, E‑Bikes und Fahrrädern über die universitäre Notaufnahme eines Level-1-Traumazentrums im Zeitraum vom 15. Juni 2019 bis 31. Oktober 2020 vorstellten, prospektiv untersucht.

**Ergebnisse:**

Es wurden 68 verunglückte E‑Scooter-Fahrer erfasst. Davon waren signifikant mehr männlich als weiblich (*p* = 0,032). Das mittlere Alter betrug 31,1 (±13) Jahre, und lediglich 11,8 % (*n* = 8) der Unfälle wurden polizeilich registriert. Im gleichen Zeitraum wurden 34 verunglückte E‑Bike- und 356 Fahrradfahrer erfasst. In allen 3 Gruppen waren die meisten Verletzungen im Kopfbereich, gefolgt von Verletzungen an den oberen Extremitäten. Signifikant mehr E‑Scooter-Fahrer hatten einen ISS ≥ 16 als in der Gruppe der verunglückten Fahrradfahrer (*p* = 0,016). Verunglückte E‑Scooter-Fahrer hatten eine signifikant längere Krankenhausverweildauer (KHVD) als E‑Bike-Fahrer (*p* = 0,003) und als Fahrradfahrer (*p* = 0,001). Einen Helm trugen 52,9 % (*n* = 18) der E‑Bike- und 53,3 % (*n* = 113) der Fahrradfahrer, dagegen nur 1,5 % (*n* = 1) der E‑Scooter-Fahrer. Die häufigste Unfallursache bei E‑Bike- (17,7 %; *n* = 6) und Fahrradfahrern (10,4 %; *n* = 37) war das Wegrutschen auf Straßenbahnschienen, bei E‑Scooter-Fahrern die Kollision mit einem Bordstein (7,4 %; *n* = 5).

**Diskussion:**

Die 3 untersuchten Patientenkollektive zeigten differierende Verletzungsursachen und -profile. Als Ursachen für einen erhöhten Anteil von Schwerverletzten im Vergleich zu Fahrradfahrern sind die Elektromobilität, Fahren unter Alkoholeinfluss und das unzureichende Tragen eines Helms bei E‑Scootern bei Dominanz von Kopfverletzungen zu nennen. Da 73,5 % (*n* = 50) der erfassten E‑Scooter-Unfälle nicht polizeilich registriert wurden, ist von einer sehr viel höheren Anzahl von E‑Scooter-Unfällen auszugehen als bisher angenommen. Präventive Maßnahmen könnten die Einführung einer Helmpflicht, eine höhere Anzahl von Verkehrskontrollen, Fahrsicherheitstrainings und der Ausbau von Fahrradwegen sein.

## Abstract (en)

**Background:**

The actual number of accidents in e‑scooter drivers in Germany seems to be significantly higher than the current figures from the Statistisches Bundesamt suggest. This epidemiological study examines e‑scooter injuries and compares them with e‑bike and bicycle injuries.

**Objective:**

In order to create a comparable database on the dangers of e‑scooters, e‑bikes and bicycles, the typical injury patterns were analyzed and prevention options derived from them.

**Material and methods:**

All accidents involving e‑scooters, e‑bikes and bicycles that were presented via the university emergency room of a level 1 trauma center between 15 June 2019 and 31 October 2020 were prospectively investigated.

**Results:**

In our study, 68 accidents in e‑scooter drivers were included, of which only 11.8% (*n* = 8) were recorded by the police. Significantly more of them were male than female (*p* = 0.032) with a mean age of 31.1 (±13) years. At the same time, we registered 34 accidents in e‑bike riders and 356 in cyclists. In all three groups, most injuries occurred to the head, followed by injuries to the upper extremities. Significantly more e‑scooter drivers had an ISS ≥ 16 than in the group of injured cyclists (*p* = 0.016). E‑scooter riders who had an accident had a significantly longer length of stay in hospital, than e‑bike riders (*p* = 0.003) and cyclists (*p* = 0.001), 52.9% (*n* = 18) of e‑bike riders and 53.3% (*n* = 113) cyclists wore a helmet, compared to only 1.5% (*n* = 1) of e‑scooter riders. The most common cause of accidents among e‑bike riders (17.7%; *n* = 6) and cyclists (10.4%; *n* = 37) was slipping away on tram rails, while for e‑scooter riders it was colliding with a curb (7.4%; *n* = 5).

**Conclusion:**

The three patient collectives examined showed different causes and profiles of injuries. The reasons for an increased proportion of seriously injured people compared to cyclists are electromobility, driving under the influence of alcohol and inadequate wearing of a helmet on e‑scooters when head injuries dominate. 73.5% (*n* = 50) of the e‑scooter accidents recorded by us were not registered by the police and therefore do not appear in the current statistics of the statistisches Bundesamt. As a result, a much higher number of e‑scooter accidents can be assumed. Preventive measures could include the introduction of compulsory helmets, a higher number of traffic controls and the expansion of bike tracks.

## Hintergrund und Fragestellung

In zahlreichen Ländern dominieren E(Elektro)‑Scooter als alternatives Beförderungsmittel das städtische Straßenbild [1, 17]. Sie sind dafür konzipiert, kleine Entfernungen zurückzulegen, und werden vornehmlich von Verleihfirmen über eine App zur Verfügung gestellt [23]. Beispielsweise wurden in Deutschland, den USA, Österreich oder den Niederlanden bereits E‑Scooter‑, E(Elektro)‑Bike- und Fahrradunfälle untersucht, diese jedoch nie miteinander verglichen [16, 18–20, 24]. In Deutschland wurden im Juni 2019 die ersten E‑Scooter zugelassen und so im Nahverkehr angeboten. Auf Basis der Elektrokleinstfahrzeug-Verordnung müssen E‑Scooter auf Fahrradwegen oder -streifen geführt werden; eine Helmpflicht besteht nicht. Zulassungsfähig sind Elektroroller bis maximal 20 km/h [10]. E‑Bikes werden in Pedelecs und S‑Pedelecs unterschieden. Pedelecs sind Elektrofahrräder, die mit Muskelkraft sowie durch einen Hilfsmotor, der die Pedalleistung unterstützt, betrieben werden. Die Höchstgeschwindigkeit ist hier auf 25 km/h beschränkt. Eine gesonderte Versicherungspflicht oder Helmpflicht besteht hier nicht. S‑Pedelecs haben dagegen eine Höchstgeschwindigkeit von 45 km/h und gehören zu den Kleinkrafträdern. Hierfür sind ein Mofaführerschein, ein Versicherungskennzeichen sowie eine Helmpflicht vorgeschrieben [10].

Entgegen der gesetzlichen Verordnung sind die typischen Verletzungsmuster bei Unfällen mit E‑Scooter-Beteiligung v. a. im Unterschied zu anderen alternativen Beförderungsmitteln im Straßenverkehr, wie dem E‑Bike oder dem Fahrrad, nicht hinreichend untersucht. Zum einen soll diese Studie eine wissenschaftlich begründete Diskussionsgrundlage für den Vergleich der 3 Beförderungsmittel liefern. Zum anderen sollen Empfehlungen zur möglichen Unfallprävention für eine sichere Benutzung von E‑Scootern abgeleitet werden.

## Studiendesign und Untersuchungsmethoden

An der Klinik für Unfall‑, Hand- und Wiederherstellungschirurgie des Universitätsklinikums Essen wurden im Zeitraum vom 15. Juni 2019 bis 31. Oktober 2020 prospektiv Unfälle mit Beteiligung von E‑Scootern, E‑Bikes und Fahrrädern erfasst, die sich über die Notaufnahme vorstellten. Einziges Einschlusskriterium war das Verunfallen als Fahrer eines der genannten Fortbewegungsmittel. Die erhobenen Daten (Geschlecht, Alter, Aufnahmedatum, Unfalldatum, Unfallzeitpunkt, Aufnahme mit RTW(Rettungswagen)/Notarzt/selbstständig, Schockraum, ISS („injury severity score“), Art des Beförderungsmittels, Unfallursache, Verletzungen, Nebendiagnosen, Alkohol, Diagnostik MRT(Magnetresonanztomographie)/CT(Computertomographie)/Röntgen/Ultraschall, Art der stationären Aufnahme, Operation/Therapie, stationärer Verweildauer, BG(Berufsgenossenschaft)/Freizeit, Helm) waren Teil der klinischen Routine. Die Tageszeit wurde wie folgt definiert: 06:01 Uhr–12:00 Uhr morgens, 12:01 Uhr–18:00 Uhr nachmittags 18:01 Uhr–24:00 Uhr abends und 00:01 Uhr–06:00 Uhr nachts. *In Anlehnung an die ASA-Klassifikation wurden die Vorerkrankungen der Studienteilnehmer kategorisiert *[4]*. ASA-Klasse I entspricht in der vorliegenden Studie keinen Vorerkrankungen. ASA-Klasse II entspricht hier Vorerkrankungen mit keiner oder geringer Einschränkung des täglichen Lebens, und ASA-Klassifikation III entspricht Vorerkrankungen mit einer starken Einschränkung des täglichen Lebens. *In einem zweiten Schritt wurden alle verunglückten E‑Scooter-Fahrer per Telefonabfrage dazu befragt, ob ihr Unfall der Polizei gemeldet wurde. Die Daten wurden prospektiv anonymisiert erhoben und ausgewertet. Zur Einschätzung des Schweregrads der Verletzungen bestimmten wir den ISS zu jedem Patienten [15]. Die Studie erfolgte im Einklang mit der Deklaration von Helsinki und nach Überprüfung durch die zuständige Ethikkommission (19-8954-BO).

### Statistik

Die statistische Auswertung erfolgte mittels IBM SPSS Statistics 27 Software (Fa. IBM, Armonk, NY, USA). Deskriptive Daten wurden auf Mittelwert (M), Standardabweichung (±SD), Median (M) und Interquartilabstand (IQR) untersucht. Alle Werte wurden mittels Shapiro-Wilk-Test auf Normalverteilung geprüft. Je nachdem, ob eine Normalverteilung vorlag, wurde der *t*-Test oder der Mann-Whitney-U-Test durchgeführt. Der Zusammenhang zweier kategorialer Variablen zwischen unabhängigen Stichproben wurde mittels Chi-Quadrat-Test untersucht. Werte von *p* < 0,05 erachteten wir als signifikant.

## Ergebnisse

Insgesamt wurden 458 Patienten erfasst, davon 68 E-Scooter-Fahrer, 34 E-Bike-Fahrer und 356 Fahrradfahrer. Unterschiede in den demografischen Daten der Patienten zeigen Tab. 1, 2 und 3.

**Table 1:** 

–	Fahrzeuge									
–		E‑Scooter		E‑Bike		Fahrrad		Vergleich der Fortbewegungsmittel		
–		Anzahl (*n*)	Häufigkeit (%)	Anzahl (*n*)	Häufigkeit (%)	Anzahl (*n*)	Häufigkeit (%)	E‑Scooter vs. E‑Bike	E‑Scooter vs. Fahrrad	E‑Bike vs. Fahrrad
Helm	Unfall mit Helm	1	1,5	18	52,9	113	53,3	*p* *<* 0,001*	*p* *<* 0,001*	*p* = 0,969
	Unfall ohne Helm	67	98,5	16	47,1	99	46,7			
Unfallzeit	Morgens (06:01 Uhr–12:00 Uhr)	15	22,1	10	29,4	102	28,7	*p* *=* 0,043*	*p* *=* 0,011*	*p* = 0,525
	Nachmittags (12:01 Uhr–18:00 Uhr)	23	33,8	18	52,9	156	43,8			
	Abends (18:01 Uhr–24:00 Uhr)	23	33,8	6	17,6	87	24,4			
	Nachts (00:01 Uhr–06:00 Uhr)	7	10,3	0	0	11	3,1			
Unfallart	BG	19	27,9	7	20,6	82	23	*p* = 0,422	*p* = 0,384	*p* = 0,745
	Freizeit	49	72,1	27	79,4	274	77

**Table 2:** 

–	Fahrzeuge									
–		E‑Scooter		E‑Bike		Fahrrad		Vergleich der Fortbewegungsmittel		
–		Anzahl (*n*)	Häufigkeit (%)	Anzahl (*n*)	Häufigkeit (%)	Anzahl (*n*)	Häufigkeit (%)	E‑Scooter vs. E‑Bike	E‑Scooter vs. Fahrrad	E‑Bike vs. Fahrrad
Vorstellungsart	Mit Notarzt (NA)	12	17,7	7	20,6	79	22,2	*p* = 0,081	*p* = 0,552	*p* = 0,064
	Rettungswagen ohne NA	11	16,2	11	32,4	73	20,5			
	Selbstständig	45	66,2	15	44,1	203	57			
	Rettungshubschrauber (RTH)	0	0	1	2,9	1	0,3			
Verletzungsgrad	Schockraumindikation	12	17,7	9	26,5	70	19,7	*p* = 0,299	*p* = 0,700	*p* = 0,345
	ISS ≥ 16	9	13,2	6	17,7	19	5,3	*p* = 0,553	*p* *=* 0,016*	*p* *=* 0,005*
Diagnostik	Röntgen	35	51,5	15	44,1	207	58,1	*p* *=* 0,044*	*p* = 0,097	*p* = 0,161
	CT	8	11,8	2	5,9	15	4,2			
	Röntgen und CT	5	7,4	8	23,5	40	11,2			
	Schockraumdiagnostik	13	19,1	9	26,5	71	19,9			
	Nur Sonographie	0	0	0	0	2	0,6			
	Keine	7	10,3	0	0	21	5,9			
Verlauf	Stationäre Aufnahme	48	70,6	19	55,9	123	34,6	*p* *=* 0,010*	*p* = 0,411	*p* *=* 0,014*
	Intensivstation	8	16,6	10	52,6	46	37,4	*p* *=* 0,028*	*p* = 0,793	*p* *=* 0,009*
	Operation	20	29,4	13	38,2	99	27,8	*p* = 0,369	*p* = 0,788	*p* = 0,199
Vorerkrankung (VE)	VE mit starker Einschränkung des täglichen Lebens	0	0	0	0	0	0	*p* *=* 0,002*	*p* = 0,308	*p* *=* 0,006*
	VE mit geringer Einschränkung des täglichen Lebens	4	5,9	4	11,8	26	7,3			
	VE mit keiner Einschränkung des täglichen Lebens	3	4,4	9	26,4	35	9,8			
	Keine	61	89,7	21	61,8	295	82,9

**Table 3:** 

–	Fahrzeuge									
–		E‑Scooter		E‑Bike		Fahrrad		Vergleich der Fortbewegungsmittel		
–		Anzahl (*n*)	Häufigkeit (%)	Anzahl (*n*)	Häufigkeit (%)	Anzahl (*n*)	Häufigkeit (%)	E‑Scooter vs. E‑Bike	E‑Scooter vs. Fahrrad	E‑Bike vs. Fahrrad
Betroffene Körperregion	Kopf/HWS	26	38,2	12	35,3	87	24,4	*p* = 0,772	*p* *=* 0,012*	*p* = 0,130
	Wirbelsäule (ohne HWS)	0	0	3	5,9	3	0,8			
	Thorax	3	4,4	1	2,9	21	5,9			
	Abdomen	0	0	1	2,9	1	0,3			
	Obere Extremitäten	21	30,9	9	26,5	173	48,6			
	Untere Extremitäten	18	26,5	9	26,5	71	19,9			
Betroffenes Körperteil	Kopf	26	38,2	12	35,3	84	23,6	*p* = 0,276	*p* = 0,063	*p* *=* 0,013*
	HWS	0	0	0	0	4	1,1			
	Wirbelsäule (ohne HWS)	0	0	2	5,9	3	0,8			
	Thorax	3	4,4	1	2,9	21	5,9			
	Abdomen	0	0	1	2,9	1	0,3			
	Schulter/Oberarm	2	2,9	2	5,9	65	18,3			
	Ellenbogen	6	8,8	4	11,8	36	10,1			
	Unterarm/Handgelenk	6	8,8	2	5,9	30	8,4			
	Hand	7	10,3	1	2,9	41	11,5			
	Becken	0	0	0	0	4	1,1			
	Hüfte/Oberschenkel	0	0	4	11,8	0	0			
	Knie	12	17,6	2	5,9	35	9,8			
	Unterschenkel/OSG	5	7,4	3	8,8	16	4,5			
	Fuß	1	1,5	0	0	4	1,1			
Verletzungsart	Prellung/Distorsion	31	45,6	15	44,1	168	47,2	*p* = 0,955	*p* = 0,277	*p* = 0,669
	Luxation	0	0	0	0	7	2			
	RQW	11	16,2	5	14,7	34	9,6			
	Fraktur	26	38,2	14	41,2	147	41,3			

*HWS* Halswirbelsäule, *OSG* oberes Sprunggelenk, *RQW Riss-Quetsch-Wunde*

### E-Scooter-Unfälle

**Figure 1:**
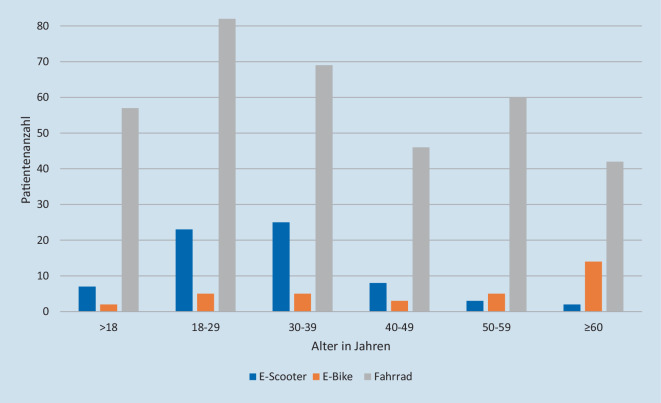

Von den 68 Patienten nach einem E‑Scooter-Unfall waren 61,8 % (*n* = 42) männlich und 38,2 % (*n* = 26) weiblich. Es verunglückten signifikant mehr männliche als weibliche Scooter-Fahrer (*p* = 0,032). Das geschlechtsübergreifende mittlere Alter der Scooter-Fahrer betrug 31,1 (±13) Jahre, bei den Männern 33,6 (±14,1) Jahre, bei den Frauen 27,2 (±10) Jahre. Die Verteilung in den Altersgruppen ist in Abb. 1 dargestellt. Durch die telefonische Abfrage konnte in der vorliegenden Studie gezeigt werden, dass 73,5 % (*n* = 50) der von uns registrierten E‑Scooter-Unfälle nicht polizeilich erfasst wurden und somit nicht in der offiziellen Statistik erfasst werden konnten. Dagegen wurden 11,8 % (*n* = 8) von der Polizei erfasst, und bei 14,7 % (*n* = 10) konnte keine Aussage darüber getroffen werden.

**Figure 2:**
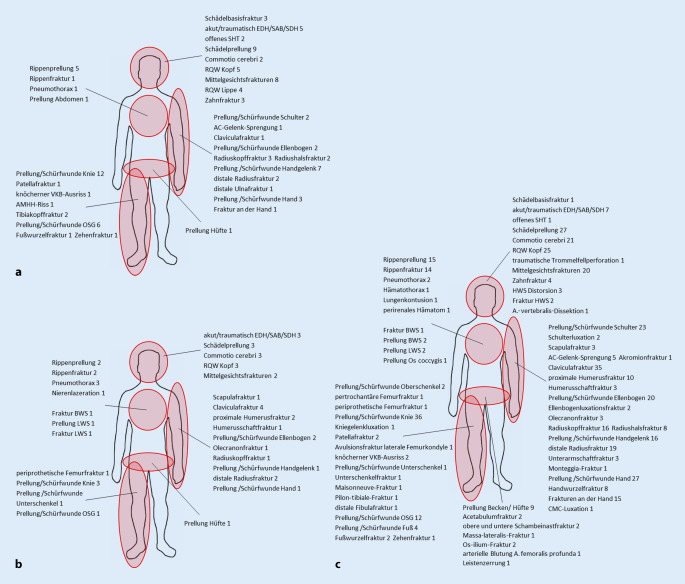

**Figure 3:**
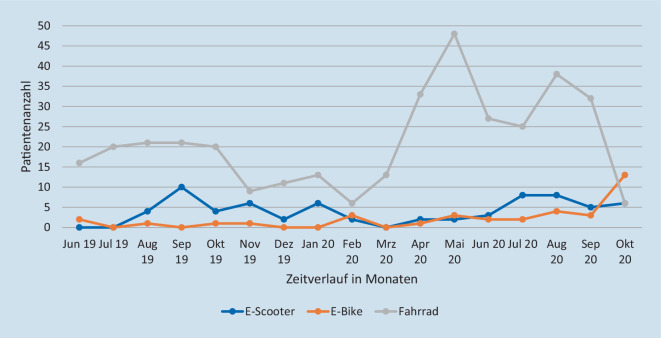

Die am häufigsten betroffene Körperregion bei Verletzungen durch einen E‑Scooter-Unfall war der Kopf/die Halswirbelsäule (Kopf/HWS) mit 38,2 % (*n* = 26), gefolgt von den oberen Extremitäten mit 30,9 % (*n* = 21) und den unteren Extremitäten mit 26,5 % (*n* = 18) (Tab. 3). Die häufigsten Verletzungen der Kopfregion waren Schädelprellungen (*n* = 9), Mittelgesichtsfrakturen (*n* = 8) und intrakranielle Blutungen (*n* = 5). Die genauen Verletzungen der einzelnen Körperregionen sind in Abb. 2 dargestellt. Nur 1,5 % (*n* = 1) der verunglückten E‑Scooter-Fahrer trugen einen Helm. Konventionell radiologische bildgebende Untersuchungen wurden in 51,5 % (*n* = 35) durchgeführt. Eine erweiterte Bildgebung mittels CT wurde in 11,8 % (*n* = 8) der Fälle indiziert. Stationär mussten 70,6 % (*n* = 48) der verletzten Scooter-Fahrer aufgenommen werden, davon 16,6 % (*n* = 8) auf eine Intensivstation, und 29,4 % (*n* = 20) wurden operiert. Die durchschnittliche stationäre Verweildauer (KHVD) nach einem E‑Scooter-Unfall betrug 3 (13) Tage. Der ISS lag bei 13,2 % (*n* = 9) bei ≥ 16. Über den Schockraum wurden 17,7 % (*n* = 12) der Patienten vorgestellt. 66,2 % (*n* = 45) stellten sich selbstständig in der Notaufnahme vor. Von den Patienten mit E‑Scooter-Unfällen hatten 89,7 % (*n* = 61) keine Vorerkrankungen. Unter Einfluss von Alkohol verunglückten 11,8 % (*n* = 8) der Patienten, dies zu 62,5 % (*n* = 5) nachts und zu 100 % (*n* = 8) an Wochenenden oder Feiertagen. 50 % (*n* = 4) gehörten zu der Gruppe der 30- bis –39-Jährigen und hatten ein mittleres Alter von 39,3 (±14) Jahren. Mit einem ISS von ≥ 16 verunglückten 50 % (*n* = 4) der alkoholisierten Scooter-Fahrer, dabei erlitten alle Kopfverletzungen. In Abb. 3 ist die Anzahl der Unfälle mit E‑Scootern im Zeitverlauf von Juni 2019 bis Oktober 2020 dargestellt. Einen ersten Peak konnten wir im Herbst 2019 und einen zweiten im Sommer 2020 verzeichnen. Während des ersten Lockdowns, im Rahmen der SARS-CoV-2-Pandemie, im Frühjahr 2020 gingen die Patientenzahlen nach E‑Scooter-Unfällen stark zurück. Nachmittags und abends ereigneten sich jeweils mit 33,8 % (*n* = 23) die meisten E‑Scooter-Unfälle. Auf dem Hin- oder Rückweg von der Arbeit ereigneten sich 27,9 % (*n* = 19) der Unfälle und wurden BGlich aufgenommen.

### E-Bike- und Fahrradunfälle

In der vorliegenden Studie waren 55,9 % (*n* = 19) der 34 verletzten E‑Bike-Fahrer männlich und 44,1 % (*n* = 15) weiblich. Das mittlere Alter betrug insgesamt 50,5 (±20,8) Jahre. Von den 356 Patienten mit Verletzungen durch einen Fahrradunfall waren 71,4 % % (*n* = 254) männlich und 28,7 % (*n* = 102) weiblich. Es zeigte sich hierbei, dass signifikant mehr männliche Fahrradfahrer verunglückten als weibliche (*p* = 0,03). Das mittlere Alter betrug insgesamt 37,1 (±18,2) Jahre. Die verunglückten E‑Bike-Fahrer waren signifikant älter als die verunglückten Fahrradfahrer (*p* < 0,001). Die genaue Verteilung in den Altersgruppen ist in Abb. 1 dargestellt. Verletzungen bei E‑Bike-Unfällen traten am häufigsten am Kopf/an der HWS mit 35,3 % (*n* = 12) auf, gefolgt von den oberen Extremitäten mit 26,5 % (*n* = 9) (Tab. 3). Die häufigsten Verletzungen der Kopfregion waren intrakranielle Blutungen (*n* = 3), Schädelprellungen (*n* = 3) und Commotio cerebri (*n* = 3), die der oberen Extremitäten Klavikulafrakturen (*n* = 4). Bei Fahrradunfällen war die am häufigsten betroffene Körperregion die obere Extremität mit 48,6 % (*n* = 173), gefolgt von Verletzungen am Kopf/an der HWS mit 24,4 % (*n* = 87) (Tab. 3). Bei Fahrradunfällen wurden besonders viele Klavikulafrakturen mit 9,8 % (*n* = 35), gefolgt von Radiuskopf- und Radiushalsfrakturen mit 6,7 % (*n* = 24) beobachtet. Die genauen Verletzungen der einzelnen Körperregionen sind in Abb. 2 dargestellt. Einen Schutzhelm trugen 52,9 % (*n* = 18) der E‑Bike- und 53,3 % (*n* = 113) der Fahrradfahrer. Selbstständig in der Notaufnahme stellten sich 44,1 % (*n* = 15) der verunglückten E‑Bike- und 57 % (*n* = 203) der verunglückten Fahrradfahrer vor. Die durchschnittliche KHVD nach einem E‑Bike-Unfall betrug 4 (5,8) Tage, nach einem Fahrradunfall 2 (3) Tage. Der ISS lag bei 17,7 % (*n* = 6) der Patienten nach einem E‑Bike-Unfall, bei 5,3 % (*n* = 19) der Patienten nach einem Fahrradunfall, bei ≥ 16. Auf einer Intensivstation mussten 52,6 % (*n* = 10) der verunglückten E‑Bike- und 37,4 % (*n* = 46) der verunglückten Fahrradfahrer überwacht werden. Über den Schockraum wurden 26,5 % (*n* = 9) der verunglückten E‑Biker vorgestellt und 19,7 % (*n* = 70) der verunglückten Fahrradfahrer. Unter Einfluss von Alkohol verunglückten 2,9 % (*n* = 1) der E‑Biker und dies nachmittags. In der Gruppe der Fahrradfahrer verunglückten 3,7 % (*n* = 13) der Fahrradfahrer unter Einfluss von Alkohol und dies mit 38,5 % (*n* = 5) meistens nachts, davon hatten 53,9 % (*n* = 7) eine Kopfverletzung und keiner trug einen Helm. Die Abb. 3 zeigt während des ersten SARS-CoV‑2 bedingten Lockdowns weniger Patienten nach einem Fahrradunfall, jedoch einen Peak bei E‑Bike-Unfällen. Die meisten E‑Bike- und Fahrradunfälle ereigneten sich nachmittags mit jeweils 52,9 % (*n* = 18) und 43,8 % (*n* = 156). Auf dem Hin- oder Rückweg von der Arbeit ereigneten sich 20,6 % (*n* = 7) der E‑Bike- und 23 % (*n* = 82) der Fahrradunfälle und wurden BGlich aufgenommen.

### Vergleich der Gruppe der verunglückten E-Scooter- mit E‑Bike- und Fahrradfahrern

Es zeigte sich, dass die E‑Scooter-Fahrer signifikant jünger waren im Vergleich zu den E‑Bikern (*p* < 0,001) und im Vergleich zu den Fahrradfahrern (*p* = 0,01). In allen 3 Gruppen waren die meisten Verletzungen am Kopf/an der HWS (E-Scooter *n* = 26, 38,2 %; E‑Bike *n* = 12, 35,3 %; Fahrrad *n* = 88, 24,7 %). Bei Unfällen mit E‑Scootern und Fahrrädern konnte ein signifikanter Zusammenhang zwischen dem Auftreten von Kopfverletzungen und dem Fahrzeugtyp festgestellt werden (*p* = 0,012). Alle Fahrer der 3 Fahrzeuggruppen erlitten nach Unfällen am häufigsten Distorsionen/Prellungen (E-Scooter *n* = 31, 45,6 %; E‑Bike *n* = 15, 44,1 %; Fahrrad *n* = 168, 47,2 %). Hier zeigen sich keine signifikanten Unterschiede zwischen den einzelnen Gruppen. Verunglückte E‑Bike-Fahrer hatten eine signifikant längere KHVD als E‑Scooter-Fahrer (*p* = 0,003) und als Fahrradfahrer (*p* = 0,001). *Die Anzahl der schwer verletzten (ISS* *≥* *16) E‑Scooter-Fahrer war signifikant höher als die der Fahrradfahrer (p* *=* *0,016). Die Anzahl an schwer verletzten E‑Bike-Fahrern war ebenfalls signifikant höher als die der Fahrradfahrer (p* *=* *0,005). Zwischen E‑Scooter-Fahrern und E‑Bike-Fahrern konnte kein signifikanter Zusammenhang bei der Anzahl an schwer Verletzten gezeigt werden (p* *=* *0,553). *Die meisten E‑Scooter-Unfälle wurden nachmittags erfasst, genau wie Fahrradunfälle und E‑Bike-Unfälle (E-Scooter *n* = 23, 33,8 %; E‑Bike *n* = 18, 52,9 %; Fahrrad *n* = 156, 43,8 %). *Ein signifikanter Unterschied konnte in der Unfallzeit zwischen verunglückten E‑Scooter-Fahrern und E‑Bike-Fahrern gezeigt werden (p* *=* *0,043). Ebenso zwischen Unfällen mit E‑Scootern und Fahrrädern (p* *=* *0,011). Bei verunglückten E‑Bike-Fahrern konnte im Vergleich zu verunglückten Fahrradfahrern kein signifikanter Unterschied gezeigt werden (p* *=* *0,525). *98,5 % (*n* = 67) der verunglückten E‑Scooter-Fahrer trugen keinen Helm. Dagegen trugen 47,1 % (*n* = 16) der E‑Biker und 46,1 % (*n* = 99) der Fahrradfahrer keinen Schutzhelm. Die häufigste Unfallursache bei Fahrradfahrern war mit 10,4 % (*n* = 37), dass das Fahrrad in oder auf Straßenbahnschienen weggerutscht sei. Selbiges wurde in der Gruppe der E‑Biker als häufigste Unfallursache mit 17,7 % (*n* = 6) angegeben. Die verunglückten E‑Scooter-Fahrer gaben als häufigste Unfallursache mit 7,4 % (*n* = 5) die Kollision mit einem Bordstein an. Das Patientenkollektiv der E‑Scooter-Fahrer zeigt sich jünger und gesünder (mittleres Alter 31,1 (±13) Jahre; mit Vorerkrankungen 10,3 %; *n* = 7) im Vergleich zu dem der Fahrradfahrer (mittleres Alter 37,1 (±18,2) Jahre; mit Vorerkrankungen 17,1 %; *n* = 61) und E‑Biker (mittleres Alter 50,5 (±20,8) Jahre; mit Vorerkrankungen 38,2 %; *n* = 13).

## Diskussion

In der vorliegenden Studie beobachteten wir die häufigsten Verletzungen bei E‑Scooter-Unfällen im Kopfbereich, gefolgt von Verletzungen an den oberen Extremitäten. Dies stimmt sowohl mit der nationalen als auch internationalen Studienlage überein, wie Daten aus deutschen Großstädten sowie den USA zeigen konnten [7, 13, 16, 21, 24]. Die eingeschlossenen Fallzahlen waren ähnlich zu der in der vorliegenden Studie. In dieser Studie verunglückten signifikant mehr männliche Scooter-Fahrer als weibliche. Das mittlere Alter der männlichen E‑Scooter-Fahrer betrug 33,6 Jahre. Auch in den national durchgeführten Studien sind mehrheitlich junge Männer Mitte 30 verunglückt [16, 24]. Das Statistische Bundesamt (Destatis) veröffentlichte am 26.03.2021 Zahlen zu E‑Scooter-Unfällen in Deutschland. Hier wurden 2155 E‑Scooter-Unfälle mit Personenschaden für ganz Deutschland in einem Jahr registriert. Für ganz Nordrhein-Westfalen waren es 566 und für Essen 21 Unfälle mit E‑Scootern [6, 11]. In der vorliegenden Studie wurden lediglich 11,8 % (*n* = 8) der von uns erfassten E‑Scooter-Unfälle polizeilich registriert. Dabei ist zu beachten, dass 73,5 % (*n* = 50) der von uns registrierten verunglückten E‑Scooter-Fahrer nicht polizeilich gemeldet wurden und nicht in der Statistik auftauchen. Folglich ist von einer sehr viel höheren Anzahl von E‑Scooter-Unfällen auszugehen, als die offizielle Statistik suggeriert. Außerdem ist von einer noch größeren Dunkelziffer an E‑Scooter-Unfällen auszugehen, da von uns nur verletzte E‑Scooter-Fahrer erfasst wurden, die sich in einer Notaufnahme vorstellten. In der vorliegenden Studie trugen lediglich 1,5 % (*n* = 1) der E‑Scooter-Fahrer einen Helm. Dies entspricht den Ergebnissen vergleichbarer Studien [2, 16, 25]. Auch in den Gruppen der hier erfassten E‑Biker und Fahrradfahrer trug nur in etwa jeder zweite (E-Bike 52,9 %, *n* = 18; Fahrrad 53,5 %, *n* = 113) einen Helm. In einer Studie von Poos et al. in der verunglückte E‑Bike- und Fahrradfahrer miteinander verglichen wurden, trug nur ein E‑Bike-Fahrer einen Helm (*n* = 92) und kein Fahrradfahrer einen Helm (*n* = 92) [20]. Aufgrund der fehlenden Helmpflicht werden kaum Schutzhelme beim Fahren eines E‑Scooters, E‑Bikes oder Fahrrads getragen, obwohl in der aktuellen Literatur mehrfach belegt wurde, dass dies die Verletzungsschwere und Mortalität der Verunglückten deutlich reduziert [3, 20, 27]. Hierdurch lässt sich die große Anzahl an Kopfverletzungen erklären. Eine Studie von Høye et al. zeigt, dass der Gebrauch eines Fahrradhelms ernsthafte Kopfverletzungen um 60 % reduzierte und dies die Mortalität von Fahrradfahrern um 34 % senken konnte [9]. In der Gruppe der Fahrradfahrer beobachteten wir oft Verletzungen an der oberen Extremität, ähnlich zu den Ergebnissen von Juhra et al. [12].

Unter Einfluss von Alkohol verunglückten in der vorliegenden Studie 11,8 % (*n* = 8) der E‑Scooter-Fahrer und dies meistens nachts. 50 % (*n* = 4) der alkoholisierten E‑Scooter-Fahrer verunglückten schwer mit einem ISS ≥ 16, und alle erlitten Kopfverletzungen. Alle alkoholbedingten E‑Scooter-Unfälle fanden an einem Wochenende oder Feiertag statt. In vergleichbaren Studien aus Deutschland war der prozentuale Anteil an alkoholisiert verunglückten E‑Scooter-Fahrern mit ca. 30 % deutlich höher. Auch hier wurden mehrheitlich verunglückte Scooter-Fahrer am Wochenende oder an Feiertagen beobachtet [16, 24]. In einer Studie aus Australien lag der Anteil an alkoholisiert verunglückten Scooter-Fahrern mit 27 % ähnlich hoch und in einer Studie aus Neuseeland sogar bei 41,9 % [5, 17]. Wir konnten zeigen, dass deutlich weniger E‑Bike- (2,9 %) und Fahrradfahrer (3,7 %) unter Einfluss von Alkohol verunglückten als E‑Scooter-Fahrer. Weitere nationale Studien bestätigten dieses Ergebnis [13]. In einer Studie aus der Schweiz wurden verunglückte E‑Biker und Fahrradfahrer miteinander verglichen. Hier zeigte sich, dass signifikant mehr Fahrradfahrer unter Alkoholeinfluss verunglückten als E‑Biker [27].

Die am häufigsten angegebene Unfallursache bei Fahrradfahrern war das Wegrutschen auf Straßenbahnschienen (10,4 %). Selbiges wurde in der Gruppe der E‑Biker am häufigsten angegeben (17,7 %). In der Gruppe der verunglückten E‑Scooter-Fahrer wurde als häufigste Unfallursache die Kollision mit einem Bordstein angegeben (7,4 %). Dies ist durch die Größe und Breite der Räder der E‑Scooter und Fahrräder bzw. E‑Bikes zu erklären. Die großen und dünnen Räder der Fahrräder und E‑Bikes bleiben öfter in den breiten Straßenbahnschienen hängen, überwinden jedoch leichter einen Bordstein. E‑Scooter-Räder bleiben nicht so leicht in Straßenbahnschienen hängen, jedoch an der Höhe der Bordsteine aufgrund der kleineren und breiteren Konfiguration. Auf die steigende Anzahl an E‑Bikes, Fahrräder und E‑Scooter sollte in der Verkehrsführung und -planung in Zukunft geachtet und reagiert werden, insbesondere mit dem Ausbau von Fahrradwegen. Der größte Anteil an verunglückten E‑Scooter‑, E‑Bike- und Fahrradfahrern stellte sich in der vorliegenden Studie selbstständig in der Notaufnahme vor. In der Studie von Störmann et al. wurden deutlich mehr Patienten durch den Rettungsdienst in der Notaufnahme vorgestellt [24]. In Nordamerika wurden ähnlich hohe Zahlen beobachtet [2, 8]. Mehrheitlich reichte ein konventionelles Röntgenbild bei den verunglückten E‑Scooter‑, E‑Bike- und Fahrradfahrern für eine Diagnostik aus. In einer Studie aus Atlanta, GA (USA) wurden ähnlich hohe Zahlen für Röntgendiagnostik (77 %) und CT-Untersuchungen (22 %) nach E‑Scooter-Unfällen erfasst [26]. Die von uns am häufigsten beobachtete Verletzungsart bei verunglückten E‑Scooter‑, E‑Bike- und Fahrradfahrern waren Prellungen/Distorsionen, gefolgt von Frakturen. Auch Kleinertz et al. beobachteten für verunglückte Fahrradfahrer mehr Prellungen (20 %) als Frakturen (9 %) [13]. Der überwiegende Teil der verunglückten E‑Scooter-Fahrer (70 %) musste stationär aufgenommen werden. Dagegen mussten deutlich weniger E‑Bike- und Fahrradfahrer nach einem Unfall stationär aufgenommen werden. Andere Studien aus Deutschland geben mit 26 % eine niedrigere Hospitalisierungsrate an, jedoch gleiche Zahlen für verunglückte E‑Scooter-Fahrer, die operiert werden mussten [24]. Die durchschnittliche KHVD nach einem E‑Scooter-Unfall betrug in der vorliegenden Studie 3 Tage, nach einem E‑Bike-Unfall 4 Tage und nach einem Fahrradunfall 2 Tage. Die Studie von Moftakhar et al. gab eine längere KHVD von im Mittel 11 Tagen für verletzte E‑Scooter-Fahrer an [18]. In unserer Studie verunglückten 13,2 % (*n* = 9) der E‑Scooter-Fahrer mit einem ISS ≥ 16. In anderen Studien aus Deutschland und Europa betrug die Anzahl an Schwerverletzten deutlich weniger [16]. In der vorliegenden Studie verletzten sich mehr E‑Bike-Fahrer mit einem ISS ≥ 16 als Fahrradfahrer. Dies ist analog zu der aktuellen Literatur, bei der E‑Bike-Fahrer im Vergleich zu Fahrradfahrer eine besondere Risikogruppe bilden und bei Unfällen schwerer verletzt werden. Dies wird dem steigenden Durchschnittsalter und der Multimorbidität der E‑Bike-Fahrer sowie den stetig wachsenden Verkaufszahlen von E‑Bikes zugeschrieben [14, 20]. Die häufigsten Verletzungen, unabhängig vom Fahrzeugtyp, ereigneten sich in unserer Studie nachmittags und an Wochenenden oder Feiertagen. Zu einem gleichen Ergebnis kamen Moftakhar et al., die einen Anstieg an Unfällen mit einem E‑Scooter am Nachmittag und frühen Abend registrieren konnten [18].

## Schlussfolgerung

Das Fahren von E‑Scootern als Nahverkehrsmittel oder in der Freizeit wird immer beliebter [22]. Es ist von einer sehr viel höheren Anzahl von E‑Scooter-Unfällen mit Personenschaden auszugehen, als bisher in der Öffentlichkeit angenommen und vom Statistischen Bundesamt veröffentlicht wurde [6]. Durch eine generelle Helmpflicht könnten die Schwere und eine Vielzahl an Verletzungen deutlich reduziert werden. Aufgrund der hohen Anzahl an verunglückten alkoholisierten E‑Scooter-Fahrern sollten mehr innerstädtische Kontrollen, v. a. an Wochenenden bzw. Feiertagen, und diese dann abends und nachts stattfinden. Durch ein besser ausgebautes Netz an Fahrradwegen, auf denen auch E‑Scooter fahren können, könnten die häufigsten Unfallursachen (Straßenbahnschienen, Bordsteine) vermieden werden. Trotz einer mutmaßlich höheren Expositionszeit beim Fahrradfahren registrierten wir schwerer verletzte E‑Scooter-Fahrer bei einer vermeintlich geringeren Expositionszeit als E‑Bike- oder Fahrradfahrer. Die vorliegende Studie unterstreicht den quantitativen Einfluss auf das Gesundheitssystem und die Dringlichkeit, Präventivmaßnahmen zu ergreifen. Dies sollte in weiteren Studien genauer erfasst werden. Eine kurze Einführung vor der Benutzung von E‑Scootern oder ein Fahrsicherheitstraining könnte den Benutzern mehr Sicherheit beim Fahren geben und als Unfallprävention wirken.

## Fazit für die Praxis

- Es ist von einer sehr viel höheren Anzahl von E‑Scooter-Unfällen auszugehen, als bisher in der Öffentlichkeit angenommen und vom Statistischen Bundesamt veröffentlicht wurde.
- Die häufigsten Verletzungen bei E‑Scooter-Unfällen sind im Kopfbereich. Eine generelle Helmpflicht wird deshalb dringlich empfohlen.
- Das Patientenkollektiv von verunglückten E‑Scooter-Fahrern ist durchschnittlich jünger als das bei Fahrrad- oder E‑Bike-Unfällen und überwiegend männlich.
- Die meisten Unfälle mit alkoholisierten E-Scooter-Fahrern ereignen sich nachts und an Wochenenden oder Feiertagen.
- Die am häufigsten angegebene Unfallursachen bei Fahrradfahrern und E‑Bikern waren das Wegrutschen auf Straßenbahnschienen und bei verunglückten E‑Scooter-Fahrern die Kollision mit einem Bordstein.
- Die häufigste Fraktur bei verunglückten Fahrradfahren waren Klavikulafrakturen und Radiuskopf‑/Radiushalsfrakturen.
- E‑Scooter-Fahrer sind im Schnitt schwerer verletzt als Fahrradfahrer.
